# Neurocognitive assessment in relation to hearing impairment and retinal neurodegeneration

**DOI:** 10.1007/s10072-025-08305-5

**Published:** 2025-06-19

**Authors:** Chan Ho Lee, Jae-Ik Kim, Kang Min Lee, Joo Hyun Park, Kunho Bae

**Affiliations:** 1 Department of Ophthalmology, Seoul National University Hospital, Seoul, South Korea; 2 Department of Ophthalmology, Dongguk University Ilsan Hospital, Goyang, Korea; 3 Department of Otorhinolaryngology-Head and Neck Surgery, Dongguk University Ilsan Hospital, Goyang, Korea

**Keywords:** Dementia, Hearing impairment, Mild cognitive impairment, Optical coherence tomography, Retinal neurodegeneration

## Abstract

**Purpose:**

Sensory impairments are significant contributors to cognitive dysfunction, but the relationship between cognitive decline and various forms of neurosensory degeneration remains poorly understood. This study aimed to evaluate retinal layer neurodegeneration and hearing impairment in the general Korean population using cognitive assessments.

**Methods:**

This cross-sectional, retrospective study included participants who underwent Optical Coherence Tomography (OCT), Pure Tone Audiometry (PTA), and the Mini-Mental State Examination (MMSE). Participants were categorized into three groups based on MMSE scores: control group (MMSE > 27), mild cognitive impairment (MCI, MMSE 23–27), and dementia group (MMSE < 23). PTA thresholds were computed using the weighted four-frequency average formula (0.5 kHz, 1 kHz, 2 kHz, and 4 kHz). OCT images were analyzed to measure the Ganglion Cell Inner Plexiform Layer (GC-IPL), Peripapillary Retinal Nerve Fiber Layer (ppRNFL), and total macular thickness. These sensory parameters were compared across the three groups.

**Results:**

A total of 196 participants were included, with a mean age of 67.0 ± 10.4 years. MMSE scores showed an inverse correlation with both age and PTA thresholds, and a positive correlation with OCT parameters (all *P* <.05). After adjusting for age, significant differences in PTA thresholds were observed across all groups. However, significant reductions in OCT parameters and best-corrected visual acuity were only seen in the dementia group compared to the control and MCI groups (all *P* <.05).

**Conclusions:**

Sensory assessments are reliable indicators of cognitive function, with hearing loss emerging as a more consistent and sensitive predictor of early functional decline than retinal thickness measurements. Advanced stages of cognitive impairment are closely linked to retinal neurodegeneration and visual impairment, underscoring the importance of careful monitoring and early intervention.

**Supplementary Information:**

The online version contains supplementary material available at 10.1007/s10072-025-08305-5.

## Introduction

The increase in the older population has brought about significant public health concerns related to disability and dependence owing to cognitive dysfunction. Cognitive decline entails deficits in cognitive processes within the brain, encompassing information acquisition, processing, storage, and retrieval [1]. This decline exists on a spectrum, from mild cognitive impairment (MCI) to dementia. The emergence of MCI can precede severe functional impairment by several years, presenting a potential preventative window to delay the clinical onset of dementia. Hence, understanding the interconnections between factors closely associated with cognitive decline is critical to predict and prevent cognitive decline and dementia amidst a rapidly aging global population.

Sensory impairment significantly contributes to cognitive dysfunction development, with hearing and vision impairment recognized as major risk factors for dementia. Age-related hearing loss, affecting about a third of the older population, is a common condition [2]. A systematic review and meta-analysis have demonstrated a significant association between age-related hearing loss and a deterioration in performance across all cognitive function domains [3].

In addition, multiple studies have explored retinal involvement evidence in cognitive dysfunction, utilizing imaging techniques like optical coherence tomography (OCT) [4]. OCT enables imaging of individual retina layers in vivo, inclusive of the ganglion cell inner plexiform layer (GC-IPL), peripapillary retinal nerve fiber layer (ppRNFL), and macular thickness and volume measurement [5–7]. Some of these studies reported variations in parameters and the extent of neurodegenerative changes observed in dementia and MCI [8, 9].

Several studies have reported an association between hearing and cognitive function, and others have reported an association between vision and cognitive function [10]. In addition, the prevalence of dual sensory impairment, a combination of vision and hearing loss, has been reported to range from approximately 5–21% [11]. However, while these prior studies have identified each category of neurosensory degeneration’s association with cognitive decline, the interrelationship between the degree of cognitive decline and the different categories of neurosensory degeneration remains inadequately understood. Therefore, this study’s main objective was to evaluate retinal layer neurodegeneration and hearing impairment within the general population based on cognitive assessments.

## Methods

### Participants

This cross-sectional study retrospectively reviewed the medical records of participants who underwent spectral domain OCT, PTA and the MMSE consecutively. These examinations were conducted at a tertiary single-center hospital between January 2005 and December 2021. The study protocol and design were approved by the Institutional Review Board of Dongguk University Ilsan Hospital, Korea, and carried out in accordance with the tenets of the Declaration of Helsinki. Before inclusion in the study, informed consent was obtained from each participant.

The study included patients who visited Dongguk University Ilsan Hospital for routine medical checkups. Among these, only those who underwent PTA and MMSE within one year before or after the OCT examination were included.

Exclusion criteria were as follows: (1) OCT of poor quality (signal strength ≤ 5) due to cataract and opacification of the visual axis. (2) Best corrected visual acuity (BCVA) less than 0.4 (Snellen). (3) Intraocular pressure (IOP) greater than 20 mmHg. (4) diabetic retinopathy, retinal vascular disease, macular degeneration, central serous chorioretinopathy, glaucoma, optic neuropathy, and other eye conditions that could influence vision and retinal microstructure, (5) a history of clinical stroke, cerebral hemorrhage, or parkinsonism, which could affect cognitive function aside from dementia, (6) a history of otologic conditions that could potentially influence audiometric results including otitis media, Meniere’s disease, tympanic membrane perforation, otosclerosis, and autoimmune inner ear disease. (7) Systolic blood pressure greater than 150 mmHg and diastolic blood pressure greater than 90 mmHg. (8) Heart disease or renal failure. (9) Systemic corticosteroid use exceeding 6 months.

### Ophthalmic examination and OCT measurement

All participants in this study underwent a comprehensive ophthalmic examination. This included BCVA, IOP, slit-lamp examination, fundus photography, and spectral domain OCT examination (CIRRUS HD-OCT 500; Carl Zeiss Meditec, Dublin, CA). OCT images were scanned by two experienced examiners using the eye tracking mode.

Two scanning protocols were employed for OCT image measurement: the macular cube protocol and the optic disc cube protocol. The macular cube protocol was performed by a 512 × 128 scan to measure macular thickness. This protocol executed 512 horizontal B-scans comprising 200 A-scans per B-scan over 1024 samplings within a 6 × 6 × 2 mm cube centered on the macula. The Early Treatment Diabetic Retinopathy Study (ETDRS) grid, consisting of three concentric circles with diameters of 1 mm, 3 mm, and 6 mm from the macular center, was used to measure the full thickness of the macula. The macular full thickness was defined as the layer between the internal limiting membrane and the outer border of the retinal pigment epithelium. The inner and outer rings of the ETDRS grid were divided into superior, inferior, temporal, and nasal quadrants, resulting in a total of nine individual sectors of the macula (see Supplementary Fig. S1).

The GC-IPL thickness was measured by the Ganglion Cell Analysis (GCA) algorithm, which identified the outer boundary of the RNFL and the outer boundary of the inner plexiform layer (IPL), providing the GC-IPL thickness. Six individual sectors were measured in an elliptical annulus around the fovea (see Supplementary Fig. S1).

The optic disc cube 200 × 200 protocol, designed to position the cube scan on the optic nerve head, was used for optic disc analysis. This protocol generated a cube of data, measuring a 6 × 6 mm grid, by scanning a series of 200 horizontal B-scans, each composed of 200 A-scans. A B-scan in the shape of a 3.46 mm diameter circle was then extracted, using the center of the optic disc as a reference point. The anterior and posterior boundaries of the RNFL were delineated, and RNFL thickness was determined at each A-scan (see Supplementary Fig. S1).

For each participant, we utilized the OCT parameters of the better eye. If the OCT parameters for the right and left eyes were similar, the right eye’s data were used.

### Pure-tone audiometry thresholds measurement

All study participants had undergone air-conduction PTA, and average thresholds (decibel hearing level, dB HL) for four PTA frequencies (0.5 kHz, 1 kHz, 2 kHz, and 4 kHz) were calculated using the weighted four-frequency average (W4FA) formula. The W4FA was formulated by dividing by the sum of the frequency thresholds ([0.5 kHz + 1 kHz + 1 kHz + 2 kHz + 2 kHz + 4 kHz]/6). By emphasizing the frequencies most important for speech understanding, W4FA provides a more accurate and functional assessment of hearing ability, which is invaluable in both clinical diagnostics and research settings [12]. For each participant in this study, we used the PTA threshold measured in the unaffected or better ear to rule out masked pathology that could affect PTA threshold. If the PTA thresholds for the right and left ears were similar (within 5 dB), the PTA threshold for the right ear was used.

### Cognitive function evaluation

The Korean version of the Mini-Mental State Examination (K-MMSE) was used to assess cognitive function. The K-MMSE consists of seven domains, scored between 0 and 30, with a lower score indicating poorer cognitive function. The K-MMSE includes thirty items providing information about orientation, attention, learning, calculation, delayed recall, and construction [13]. To analyze cognitive function alongside other parameters in this study, participants were divided into three groups (healthy control group, MCI group, and dementia group) based on their MMSE scores. Similar to methodologies employed in previous studies, participants were categorized based on their MMSE score: control group (> 27), MCI group (23–27), and dementia group (< 23) [14].

### Statistical analysis

Demographic and clinical characteristics across the three groups, classified by cognitive dysfunction, were compared using chi-square tests or one-way analysis of variance, as appropriate. Data were checked for normality using Shapiro-Wilk test. Depending on the normality, appropriate correlation analyses were performed. The Pearson correlation and multivariate regression analysis were used to assess the association of MMSE scores with age, BCVA, PTA thresholds, and OCT metrics. In multivariate regression, BCVA, and PTA thresholds were included as covariates based on their known associations with cognitive function and age. In Models 1 and 2, education level, genetic factors, and gender were considered confounders, while BCVA, PTA thresholds, macular thickness, ganglion cell-inner plexiform layer (GC-IPL) thickness, and peripapillary retinal nerve fiber layer (ppRNFL) thickness were included as explanatory variables. We also employed analysis of covariance (ANCOVA) to compare BCVA, PTA thresholds, and OCT metrics after adjusting for age. Post hoc analysis was performed using the Bonferroni method. All statistical analyses were conducted using SAS Enterprise Guide version 6.1 software (SAS Inc., Cary, NC, USA) and SPSS software version 25.0 (SPSS Inc., Chicago, IL). A P-value of less than 0.05 was considered statistically significant.

## Results

Out of the 298 participants who underwent the Mini-Mental State Examination (MMSE), OCT, and pure-tone audiometry (PTA) during the study period, 106 were excluded; 61 due to poor quality OCT images, 21 due to the presence of other retinal disorders, 10 because of glaucoma, 6 owing to a previous history of clinical stroke, cerebral hemorrhage, or parkinsonism, and 4 because of otologic diseases, 4 due to insufficient systemic general data. Consequently, 192 participants were included in the analysis. The average age was 67.0 ± 10.4 years. The baseline demographic and ocular characteristics of the study participants are presented (Table 1). According to the MMSE score classification, 95 participants (49.5%) were categorized into the MCI group, 55 participants (28.6%) into the dementia group, and 42 participants (21.9%) were deemed healthy controls. Significant differences were observed between the groups in terms of average age and visual acuity, with the dementia group being the oldest (*P* =.001) and possessing the worst BCVA (*P* <.001) (Table 1).

**Table 1:** Baseline characteristics and demographics of participants in control, MCI group and dementia group

Parameters	Total (*n* = 192)	Control (*n* = 42)	MCI (*n* = 95)	Dementia (*n* = 55)	*P* value
Age, mean (SD), years	67.0 (10.4)	62.6 (12.2)	66.9 (10.2)	70.6 (8.2)	0.001*
Diabetes mellitus, N (%)	67 (34.9)	18 (42.9)	36 (37.9)	13 (23.6)	0.099†
Hypertension, N (%)	108 (56.3)	28 (66.7)	48 (50.5)	32 (58.2)	0.202†
Male, N (%)	83 (43.2)	20 (47.6)	45 (47.4)	18 (32.7)	0.177†
Phakia, N (%)	100 (52.1)	25 (59.5)	53 (55.8)	22 (40.0)	0.097†
IOP, mean (SD), mmHg	12.1 (3.1)	12.8 (3.4)	12.2 (2.9)	11.3 (3.1)	0.089*
BCVA, logMAR, mean (SD)	0.08 (0.10)	0.02 (0.05)	0.07 (0.08)	0.14 (0.13)	< 0.001*
Reflective error, mean (SD), D	0.28 (1.85)	0.04 (1.38)	0.32 (2.17)	0.41 (1.54)	0.601*
PTA threshold, mean (SD), dB HL	32.4 (16.4)	17.6 (8.3)	31.3 (15.7)	43.7 (13.2)	< 0.001*
Hearing aid, N (%)	18 (9.4)	2 (4.8)	7 (7.4)	9 (16.4)	0.097†
BMI, mean (SD), kg/m^2^	24.7 (3.6)	24.9 (3.1)	25.0 (3.7)	24.1 (3.4)	0.353*
Initial SBP, mean (SD), mmHg	129.9 (13.8)	129.7 (14.1)	129.2 (14.3)	131.2 (13.3)	0.700*
Initial DBP, mean (SD), mmHg	73.8 (9.9)	73.3 (9.6)	74.5 (9.8)	73.1 (10.5)	0.641*
Total cholesterol, mean (SD), mg/dL	160.3 (42.0)	158.6 (35.6)	160.6 (44.7)	161.2 (42.5)	0.951*
Initial BUN, mean (SD), mg	17.1 (7.3)	16.5 (5.7)	17.7 (8.0)	16.7 (7.3)	0.597*
Initial Creatine, mean (SD), mg/dL	0.93 (0.54)	0.75 (0.18)	0.97 (0.46)	0.99 (0.79)	0.055*

Abbreviations: MCI, Mild cognitive impairment; SD, Standard deviation; IOP, Intraocular pressure; BCVA, Best corrected visual acuity; PTA, Pure tone audiometry; HL, hearing level; BMI, Body max index; SBP, Systolic blood pressure; DBP, Diastolic blood pressure; BUN, Blood urea nitrogen

Mean thresholds of PTA frequencies were calculated using the weighted four-frequency average formula ([0.5 kHz + 1 kHz + 1 kHz + 2 kHz + 2 kHz + 4 kHz]/6)

*P value by one-way analysis of variance; †P value by Chi-square test. (*P* <.05)

Normality assumptions were met, and Pearson correlation analysis revealed significant negative correlation was found between MMSE scores and age (*P* <.001), PTA threshold (*P* <.001), and BCVA (*P* <.001) (Table 2). The MMSE scores significantly and positively correlated with all retinal layer thicknesses (OCT parameters), including total macular thickness, GC-IPL thickness, and ppRNFL thickness (all *P* <.05) (Table 2).

**Table 2:** Correlation coefficients for the associations of MMSE score with age, IOP, BCVA, PTA threshold and OCT metrics index

Parameters	MMSE score	
	*r*	*P* value
Age (years)	-0.301	< 0.001
IOP (mmHg)	0.178	0.013
BCVA, logMAR	-0.448	< 0.001
PTA threshold (dB HL)	-0.553	< 0.001
**Macular thickness (um)**		
Central	0.371	< 0.001
Inner superior	0.449	< 0.001
Inner inferior	0.261	< 0.001
Inner temporal	0.253	< 0.001
Inner nasal	0.271	< 0.001
Outer superior	0.202	0.005
Outer inferior	0.306	< 0.001
Outer temporal	0.237	0.001
Outer nasal	0.252	< 0.001
**GC-IPL thickness (um)**		
Superior	0.369	< 0.001
Inferior	0.324	< 0.001
Supero-temporal	0.250	< 0.001
Supero-nasal	0.278	< 0.001
Infero-temporal	0.341	< 0.001
Infero-nasal	0.286	< 0.001
Average	0.430	< 0.001
Minimum	0.399	< 0.001
**ppRNFL thickness (um)**		
Superior	0.298	< 0.001
Inferior	0.379	< 0.001
Temporal	0.225	0.002
Nasal	0.238	0.001

Abbreviations: MMSE, Mini-mental state examination; IOP, Intraocular pressure; BCVA, Best corrected visual acuity; PTA, Pure tone audiometry; HL, hearing level; GC-IPL, Ganglion cell - inner plexiform layer; ppRNFL, peripapillary Retinal nerve fiber layer

**Fig. 1:**
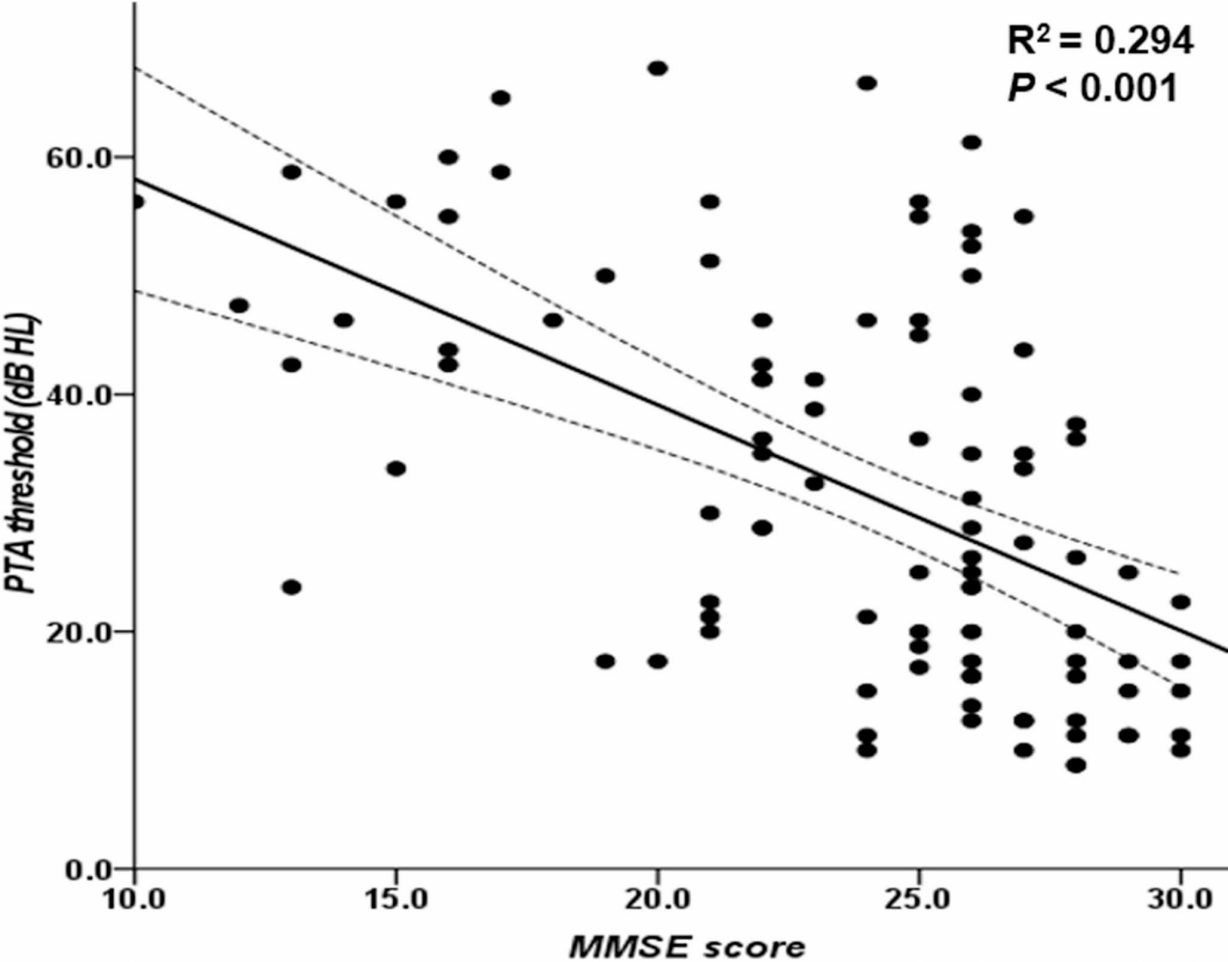
This figure presents the multiple linear regression analysis between the Pure Tone Audiometry (PTA) threshold and Mini-Mental State Examination (MMSE) scores. A significant negative correlation is observed between the mean PTA threshold and MMSE score (*P* <.001). The bold black line denotes the mean, and the dotted lines above and below extend to the upper and lower values of the 95% confidence interval, respectively

After adjusting for age, PTA threshold and BCVA were still negatively correlated with MMSE scores (*P* <.001, *P* =.001, respectively) (Table 3; Fig. 1). Foveal thickness (*P* =.046), inner superior macular thickness (*P* =.002), and inferior ppRNFL thickness (*P* <.001) were also found to be positively correlated with MMSE scores (Table 3).

**Table 3:** Multiple linear regression analysis for MMSE score with independent parameters

Independent parameters	B (Standardized)	S.E	*P* value	Adjusted *R*^2^ (*P* value)
Total (*N* = 192)				0.610 (< 0.001)
IOP (mmHg)	0.329 (0.214)	0.124	0.010	
BCVA, logMAR	-8.608 (-0.263)	2.538	0.001	
PTA threshold (dB HL)	-0.103 (-0.364)	0.024	< 0.001	
**Macular thickness (um)**				
Fovea	0.026 (0.154)	0.014	0.046	
Inner superior	0.075 (0.277)	0.023	0.002	
**GC-IPL thickness (um)**				
Supero-temporal	-1.544 (-2.795)	0.761	0.046	
Supero-nasal	-1.563 (-2.720)	0.750	0.041	
**ppRNFL thickness (um)**				
Inferior	0.060 (0.284)	0.018	< 0.001	

Abbreviations: MMSE, Mini-mental state examination; S.E, Standard error; IOP, Intraocular pressure; BCVA, Best corrected visual acuity; PTA, Pure tone audiometry; HL, hearing level; ppRNFL, peripapillary Retinal nerve fiber layer

**Fig. 2:**
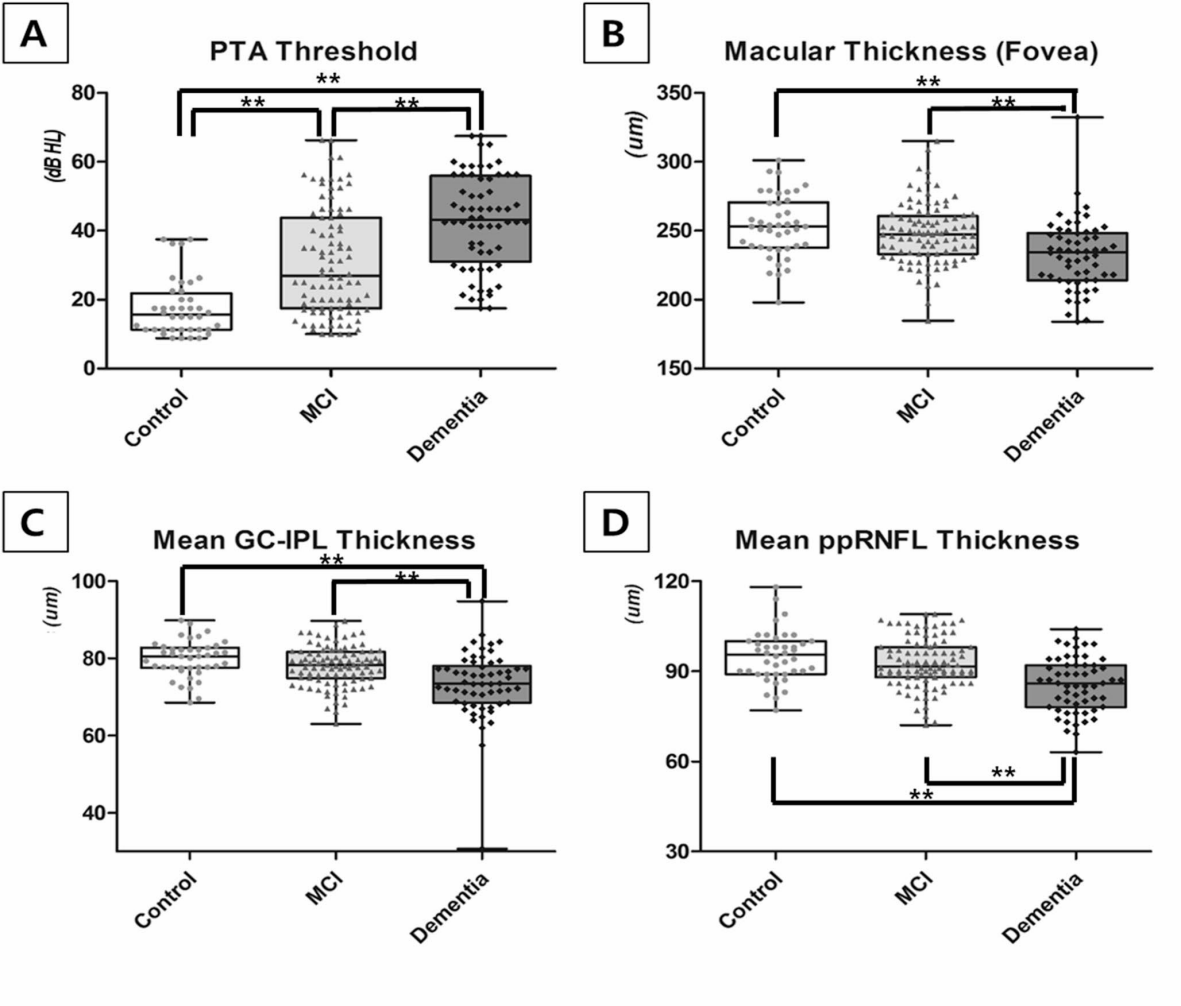
This figure compares the status of neurosensory impairment between the three groups: control, Mild Cognitive Impairment (MCI), and dementia. The distributions of PTA threshold **(A)**, mean foveal thickness **(B)**, mean Ganglion Cell Inner Plexiform Layer (GC-IPL) thickness **(C)**, and mean peripapillary Retinal Nerve Fiber Layer (ppRNFL) thickness **(D)** are displayed in relation to cognitive decline status. The black line represents the mean, and the rectangle’s upper and lower edges extend to the superior and inferior values of the 95% confidence interval respectively

An age-adjusted Bonferroni’s post hoc analysis was performed to identify parameters statistically different between the subgroups (Table 4). The MCI group had a significantly higher PTA threshold compared to the control group (*P* =.024), yet differences in OCT parameters between these two groups were not statistically significant (all *P* >.05). The dementia group had significantly worse BCVA (*P* =.015) and higher PTA thresholds (*P* =.001) compared to the MCI group. Furthermore, OCT parameters including macular thickness, GC-IPL thickness, and ppRNFL thickness were significantly thinner in the dementia group compared to the MCI or control group (all *P* <.05). The only variable demonstrating a significant difference across all three groups - control, MCI, and dementia - was the PTA threshold (all *P* <.05) (Fig. 2). Nonetheless, the dementia group exhibited thinning of retinal layers, such as inner and outer macular thickness, sectoral GC-IPL thickness, and sectoral ppRNFL thickness, compared to the other groups.

**Table 4:** Age-adjusted bonferroni’s post hoc analysis between subgroups

Parameters	Control (*N* = 42)	MCI (*N* = 95)	Dementia (*N* = 55)	*P* values		
				Control vs. MCI	MCI vs. Dementia	Control vs. Dementia
IOP (mmHg)	12.8 (3.4)	12.2 (2.9)	11.3 (3.1)	0.995	0.714	0.403
BCVA, logMAR	0.02 (0.05)	0.07 (0.08)	0.14 (0.13)	0.202	0.015	0.001
PTA threshold (dB HL)	17.6 (8.3)	31.3 (15.7)	43.7 (13.2)	0.024	0.001	< 0.001
**Macular thickness (um)**						
Fovea	253.1 (22.5)	247.9 (22.3)	232.3 (25.6)	0.525	< 0.001	< 0.001
Inner superior	319.4 (14.2)	315.2 (14.6)	301.2 (20.4)	0.824	< 0.001	< 0.001
Inner inferior	316.2 (15.9)	311.9 (15.5)	304.1 (16.7)	0.727	0.017	0.002
Inner temporal	308.3 (17.1)	305.3 (13.8)	298.4 (15.6)	0.133	0.020	0.011
Inner nasal	322.9 (17.5)	317.3 (15.8)	307.3 (19.2)	0.422	0.001	< 0.001
Outer superior	280.6 (14.2)	277.1 (14.6)	270.9 (14.4)	0.726	0.024	0.003
Outer inferior	268.6 (13.1)	263.9 (16.0)	255.8 (16.5)	0.704	0.020	0.002
Outer temporal	263.6 (13.9)	260.9 (14.4)	255.3 (13.5)	1.000	0.041	0.028
Outer nasal	295.0 (14.7)	292.0 (18.0)	285.3 (15.8)	1.000	0.038	0.030
**GC-IPL thickness (um)**						
Superior	81.1 (6.5)	78.9 (6.7)	72.6 (12.4)	0.974	< 0.001	< 0.001
Inferior	77.5 (6.1)	75.7 (9.0)	72.4 (9.5)	1.000	0.037	0.032
Supero-temporal	78.9 (5.4)	77.7 (6.0)	75.0 (8.0)	1.000	0.011	0.012
Supero-nasal	81.3 (7.2)	79.2 (7.8)	75.8 (10.6)	0.841	0.020	0.004
Infero-temporal	80.8 (6.0)	78.7 (8.0)	74.6 (7.8)	0.727	0.010	0.002
Infero-nasal	79.6 (9.1)	78.0 (8.2)	73.7 (8.0)	1.000	0.006	0.008
Average	79.9 (4.8)	78.2 (5.1)	74.0 (6.9)	0.743	< 0.001	< 0.001
Minimum	73.2 (8.1)	70.3 (8.2)	65.8 (11.4)	0.736	0.007	0.002
**ppRNFL thickness (um)**						
Superior	117.9 (15.3)	113.4 (14.1)	106.5 (14.7)	0.652	0.011	0.002
Inferior	122.7 (17.3)	119.9 (17.8)	107.6 (20.5)	0.189	< 0.001	0.001
Temporal	70.4 (11.0)	68.4 (9.4)	64.2 (9.1)	0.854	0.007	0.003
Nasal	68.6 (7.3)	68.5 (8.5)	64.5 (8.6)	1.000	0.010	0.038

Abbreviations: MCI, mild cognitive impairment; IOP, intraocular pressure; BCVA, best corrected visual acuity; PTA, pure tone audiometry; HL, hearing level; GC-IPL, Ganglion cell-inner plexiform layer; ppRNFL, peripapillary retinal nerve fiber layer

*All values were expressed as mean (standard deviation).*

Table 5 illustrates univariate and multivariate logistic regression analysis data with age correction. Model 1 assessed the association between the control and MCI groups, using the control group as a reference. In the first model’s multivariate regression, higher PTA thresholds (Odds ratio, OR = 1.13, 95% confidence interval, CI = 1.04–1.22; *P* =.006) were associated with the presence of MCI. The second model evaluated the association between the MCI group and the dementia group, using the MCI group as a reference. In the second model’s multivariate regression, lower BCVA (OR = 0.91, 95% CI = 0.83–0.99; *P* =.042), higher PTA thresholds (OR = 1.14, 95% CI = 1.00-1.29; *P* =.045), and decrease of central macular (foveal) thickness (OR = 1.04, 95% CI = 0.98–1.09; *P* =.043) and inferior ppRNFL thickness (OR = 1.16, 95% CI = 1.03–1.29; *P* =.032) were associated with the presence of dementia.

**Table 5:** Univariate and multivariate analyses of the association of visual acuity, PTA threshold, retinal layer thickness (macula, GC-IPL, ppRNFL) and systemic conditions with the presence of MCI (1st model) and the presence of dementia (2nd model). BCVA, and PTA thresholds were included as covariates based on their known associations with cognitive function and age. (*P* <.05)

Variable	1st Model: Control (Reference) vs. MCI				2nd Model: MCI (Reference) vs. Dementia			
	Univariate		Multivariate		Univariate		Multivariate	
	OR (95% CI)	*P* -value	OR (95% CI)	*P* -value	OR (95% CI)	*P* -value	OR (95% CI)	*P* -value
BCVA (Snellen) **(Per 0.01 increase)**	0.93 (0.89–0.97)	0.001	0.86 (0.71–1.01)	0.073	0.97 (0.95–0.99)	0.001	0.91 (0.83–0.99)	0.042
PTA threshold (dB HL)	1.10 (1.03–1.17)	0.004	1.13 (1.04–1.22)	0.006	1.06 (1.02–1.09)	0.001	1.14 (1.00-1.29)	0.045
Diabetes mellitus	1.25 (0.60–2.61)	0.553			1.97 (0.93–4.16)	0.075		
Hypertension	0.52 (0.25–1.11)	0.091			1.36 (0.70–2.66)	0.366		
BMI (kg/m^2^)	1.01 (0.91–1.12)	0.849			0.94 (0.85–1.03)	0.174		
Initial SBP (mmHg)	0.86 (0.97–1.02)	0.998			1.01 (0.99–1.04)	0.400		
Initial DBP (mmHg)	1.01 (0.98–1.05)	0.455			0.99 (0.95–1.02)	0.399		
Total cholesterol (mg/dL)	1.00 (0.99–1.01)	0.727			1.00 (0.99–1.01)	0.930		
Initial BUN (mg)	1.02 (0.97–1.08)	0.383			0.98 (0.94–1.03)	0.475		
Initial Creatine (mg/dL) **(Per 0.1 increase)**	1.54 (0.24–2.84)	0.004	2.73 (0.29–5.07)	0.016	1.03 (0.60–1.79)	0.907		
**Macular Thickness (um) (Per 1 μm decrease)**								
Fovea	1.01 (1.00-1.03)	0.202			1.03 (1.01–1.05)	0.001	1.04 (0.98–1.09)	0.043
Inner superior	1.02 (0.99–1.04)	0.121			1.05 (1.03–1.07)	< 0.001		
Inner inferior	1.02 (1.00-1.04)	0.113			1.03 (1.01–1.05)	0.006		
Inner temporal	1.01 (0.99–1.04)	0.230			1.03 (1.01–1.06)	0.008		
Inner nasal	1.02 (1.00-1.04)	0.072			1.03 (1.01–1.05)	0.001	1.08 (0.99–1.16)	0.087
Outer superior	1.02 (0.99–1.04)	0.168			1.03 (1.01–1.05)	0.016		
Outer inferior	1.02 (0.99–1.04)	0.088			1.03 (1.01–1.05)	0.006		
Outer temporal	1.01 (0.98–1.04)	0.313			1.03 (1.00-1.05)	0.023	1.08 (0.99–1.16)	0.081
Outer nasal	1.01 (0.99–1.03)	0.329			1.02 (1.00-1.04)	0.027		
**GC-IPL thickness (um) (Per 1 μm decrease)**								
Superior	1.05 (0.98–1.10)	0.063			1.08 (1.03–1.12)	< 0.001		
Inferior	1.01 (0.97–1.06)	0.295			1.04 (1.00-1.07)	0.037		
Supero-temporal	1.03 (0.96–1.10)	0.247			1.05 (1.00-1.10)	0.026		
Supero-nasal	1.04 (0.99–1.09)	0.141			1.04 (1.00-1.08)	0.035		
Infero-temporal	1.04 (0.99–1.09)	0.133			1.06 (1.02–1.10)	0.005		
Infero-nasal	1.02 (0.98–1.07)	0.323			1.07 (1.02–1.10)	0.002		
Average	1.07 (1.00-1.14)	0.054			1.11 (1.05–1.16)	< 0.001		
Minimum	1.05 (1.00-1.09)	0.061			1.05 (1.01–1.08)	0.008		
**ppRNFL thickness (um) (Per 1 μm decrease)**								
Superior	1.02 (1.00-1.05)	0.100			1.03 (1.01–1.06)	0.006		
Inferior	1.01 (0.99–1.03)	0.388			1.03 (1.01–1.05)	< 0.001	1.16 (1.03–1.29)	0.032
Temporal	1.02 (0.98–1.05)	0.293			1.05 (1.01–1.08)	0.011		
Nasal	1.00 (0.96–1.05)	0.934			1.05 (1.01–1.09)	0.008		

Abbreviations: MCI, Mild cognitive impairment; OR, Odds ratio; CI, Confidence interval; BCVA, best corrected visual acuity; PTA, pure tone audiometry; HL, hearing level; BMI, Body max index; SBP, Systolic blood pressure; DBP, Diastolic blood pressure; BUN, Blood urea nitrogen; GC-IPL, Ganglion cell-inner plexiform layer; ppRNFL, peripapillary Retinal nerve fiber layer

## Discussion

The cognitive function of the study participants, as evaluated by MMSE scores, demonstrated a substantial negative correlation with PTA thresholds and a positive correlation with OCT parameters, including central foveal thickness, macular GC-IPL, and ppRNFL thickness. When comparing the three groups classified by MMSE score, all groups displayed significant differences in PTA. However, only the patients with advanced cognitive decline (the dementia group) showed notable decreases in OCT parameters and visual acuity. These findings suggest that the extent of vulnerability and degeneration of the neurosensory system varies with the degree of cognitive dysfunction.

A postmortem study revealed an association between retinal ganglion cell loss in the macula and Alzheimer’s disease [15]. Pathologic examinations of retinas from Alzheimer’s disease patients uncovered Aß plaques in the inner retina, particularly in the retinal GC-IPL. This suggests that the deposition of Aß plaques may instigate inflammation and lead to macular GC-IPL degeneration. Additionally, 97% of GC-IPL neurons in the central region of the retina are ganglion cells, implying that the thickness of the central retinal layer may be a useful indicator for assessing neurodegenerative changes in dementia [15]. Based on these findings, previous studies reported a significant association between the thickness of specific layers (GC-IPL, RNFL) or total macular thickness and the presence of dementia, suggesting that OCT could serve as a valuable imaging tool for identifying individuals at high risk of dementia [16–18]. In line with previous studies, the current study found that the presence of dementia was associated with total macular thickness (central and foveal), macular GC-IPL thickness, and inferior ppRNFL thickness.

Before the retinal changes were reported, studies had already demonstrated a close relationship between hearing and cognitive function. Magdalene et al. reported that hearing loss during the early stages of neurocognitive decline was significantly associated with lower cognitive function [19]. This study found a 2.8% decrease in MMSE scores for every 10 dB HL of hearing loss [20]. Other research has indicated a correlation between hearing loss and rapid declines in MMSE scores, especially in cases of moderate and severe hearing loss [21]. Several hypotheses have been proposed to explain this correlation. One theory suggests the link between hearing impairment and cognitive impairment might lie in the medial temporal cortex due to the close anatomical relationship of auditory and vestibular structures. This cortex is known to play a significant role in cognition [22]. Another theory, known as the “cognitive load hypothesis”, proposes that because the brain is constantly attempting to understand words and sounds in patients with hearing impairment, it becomes overworked and less efficient, resulting in a decrease in cognitive function. The “cascade hypothesis”, on the other hand, suggests that impairments linked to hearing loss could directly or indirectly cause brain atrophy, leading to cognitive impairment [23]. Despite considerable research, the underlying causal relationship between auditory and cognitive decline remains unclear. However, a large-scale study using the UK Biobank dataset found that hearing aid use was associated with better cognition. Furthermore, in a long-term follow-up study, hearing aid use was reported to mitigate age-related cognitive decline, suggesting a strong interconnection between hearing and cognition [24, 25].

Neurosensory degeneration, cognitive decline, and aging are intricately related, yet the complex interrelationships among these factors remain to be fully understood. In the present study, neurosensory degeneration varied depending on cognitive function status after adjusting for age. Specifically, hearing appears to be impacted from the early stage of cognitive decline, while degenerative changes in the retina become significantly apparent at a more advanced stage of cognitive decline. Subtle changes in the retina (such as retinal layer thinning) or mild visual impairment may cause difficulties like distinguishing small letters or slowing reading speed, but these typically have a limited effect on social interactions such as conversation. Conversely, even early hearing loss can lead to communication problems that potentially weaken social bonds or even result in depression [26]. This implies that among the neurosensory system’s degenerative changes, those caused by hearing loss might affect daily personal relationships earlier [27]. This social isolation is linked to, and can worsen, cognitive decline [28, 29]. In addition, we also identified potential synergistic effects of sensory impairment on cognitive decline. Exploring these interactions in the future may provide deeper insights into the mechanisms underlying cognitive dysfunction in individuals with multiple sensory impairment.

Several studies have noted the association of retinal degenerative change with the advanced stage of cognitive decline. For instance, in a study by Ito et al. investigating the correlation between OCT parameters and MMSE score, GC-IPL and macular thicknesses were thinner in dementia, but MCI was not associated with any OCT measurements [17]. Similarly, in a study by Yoon et al., no significant differences in GC-IPL thickness were found between MCI subjects and controls, but a significantly reduced GC-IPL thickness was observed in dementia compared to MCI and controls [30, 31]. Several mechanisms have been postulated to support these complex interrelationships. In patients with dementia, damage to brain regions encompassing the visual tract might cause retrograde degeneration of the optic nerve by affecting the neuronal connections of the visual tract [5]. This retrograde degeneration of the visual tract and subsequent optic nerve damage can cause swelling and gliosis formation in the axons. In reality, there seems to be a delay between optic nerve degeneration and retinal ganglion cell loss, during which swelling or gliosis formation can occur, followed by more evident structural losses [31, 32]. By these mechanisms, neurosensory retina deterioration might present at a relatively late stage.

This study has several limitations. First, being a single-center, cross-sectional study, which may limit the generalizability of the results, and it did not address longitudinal change. Secondly, given the retrospective nature of the study, the examinations could not be performed simultaneously. However, to minimize bias, we included only patients who underwent all examinations within a similar time period. Thirdly, we classified the groups using an arbitrary MMSE value cutoff, which is a common approach in prior studies. Fourthly, hearing and vision deterioration itself can affect the MMSE score, a test that evaluates cognitive function, and this bias is a limitation. Fifth, retinal thickness and hearing function naturally decrease with aging, and although there was a significant difference in age between groups in this study, these results remained significant after adjusting for age in multivariate analysis. Despite these limitations, this study benefits from identifying the interrelationship of various categories of neurosensory degeneration with cognitive dysfunction in a general population that underwent a routine health check-up.

In conclusion, sensory measures are generally reliable predictors of cognitive function. Notably, hearing loss is a more consistent and better predictor of age-related functional decline from an early stage than retinal thickness measures. Retinal neurodegenerative changes occur in advanced stages of cognitive impairment and are closely associated with visual impairment. This contributes to our understanding of how cognitive decline impacts neurosensory impairment, and aids in the early detection of cognitive dysfunction to mitigate disability and dependency. Further longitudinal studies may enhance our understanding of the temporal antecedent changes between cognitive and neurosensory functions.

## Electronic supplementary material

Below is the link to the electronic supplementary material.

Supplementary Material 1: Figure S1. Representative image showing the method for measuring of optical coherence tomography parameters. (**A**) The thickness of the macula in each sector (Fovea [F], inner superior [IS], inner inferior [II], inner temporal [IT], inner nasal [IN], outer superior [OS], outer inferior [OI], outer temporal [OT], outer nasal [ON]). (**B**) Cross sectional image of macula. Macular thickness was automatically segmented by the inbuilt software. (**C**) The thickness of the ganglion cell inner plexiform layer (GC-IPL) in each sector (Superior [Sup], inferior [Inf], supero-temporal [ST], supero-nasal [SN], infero-temporal [IT], infero-nasal [IN]) was automatically measured by the ganglion cell analysis algorithm by the inbuilt software. (**D**) Cross sectional image of macula. GC-IPL thickness was automatically segmented by the inbuilt software. (**E**) Peripapillary retinal nerve fiber layer (ppRNFL) thickness of each sectors (Superior [S], inferior [I], temporal [T], nasal [N]) was obtained from the disc cube scan using the same method. (**F**) Cross sectional image of ppRNFL. ppRNFL thickness was automatically segmented by the inbuilt software

## Data Availability

The datasets used and/or analysed during the current study available from the corresponding author on reasonable request.

## Declarations

### Statistical assistance

Statistical consultation was provided by Ju-Yeun Lee, M.D., Ph.D., Department of Preventive Medicine, Seoul National University.
