# Supplementary figures and images for: Neurocognitive assessment in relation to hearing impairment and retinal neurodegeneration
Source: Neurol Sci. 2025 Jun 19;46(9):4309–20. doi: 10.1007/s10072-025-08305-5 (PMC12394375; doi:10.1007/s10072-025-08305-5)

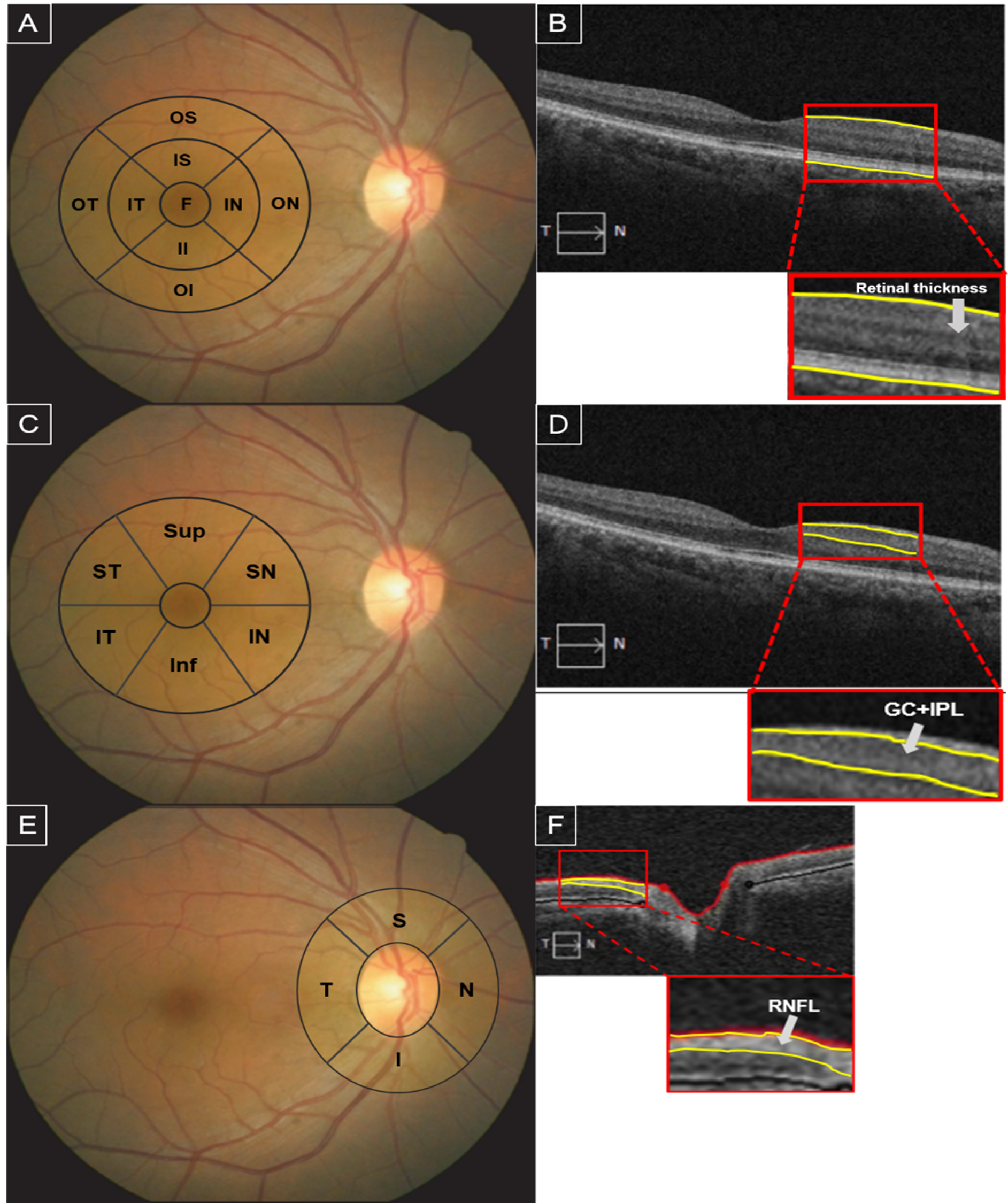

Supplement: Supplementary file 1 — Supplementary Material 1: Figure S1. Representative image showing the method for measuring of optical coherence tomography parameters. (A) The thickness of the macula in each sector (Fovea [F], inner superior [IS], inner inferior [II], inner temporal [IT], inner nasal [IN], outer superior [OS], outer inferior [OI], outer temporal [OT], outer nasal [ON]). (B) Cross sectional image of macula. Macular thickness was automatically segmented by the inbuilt software. (C) The thickness of the ganglion cell inner plexiform layer (GC-IPL) in each sector (Superior [Sup], inferior [Inf], supero-temporal [ST], supero-nasal [SN], infero-temporal [IT], infero-nasal [IN]) was automatically measured by the ganglion cell analysis algorithm by the inbuilt software. (D) Cross sectional image of macula. GC-IPL thickness was automatically segmented by the inbuilt software. (E) Peripapillary retinal nerve fiber layer (ppRNFL) thickness of each sectors (Superior [S], inferior [I], temporal [T], nasal [N]) was obtained from the disc cube scan using the same method. (F) Cross sectional image of ppRNFL. ppRNFL thickness was automatically segmented by the inbuilt software [file 10072_2025_8305_MOESM1_ESM.tif]
